# The interactive use of augmented reality for educating the elderly on common age-related eye disease

**DOI:** 10.1186/s12877-024-05658-y

**Published:** 2025-01-03

**Authors:** Yuet Yi Hung, Wang Yee Chu, Juming Jiang, Wing Yiu Yeung, Wing Huen Yan, Tai On Kwok, Yau Kei Chan

**Affiliations:** 1 LKS Faculty of Medicine, The University of Hong Kong, Pokfulam, Hong Kong; 2 Department of Ophthalmology, LKS Faculty of Medicine, The University of Hong Kong, Pokfulam, Hong Kong; 3 School of Nursing and Health Studies, Hong Kong Metropolitan University, Ho Man Tin, Hong Kong; 4 Room 301, Level 3, Block B, Cyberport 4, 100 Cyberport Road, Telegraph Bay, Hong Kong

**Keywords:** Augmented reality, Visual health, Elderly, Ophthalmic education, Age-related eye disorders

## Abstract

**Background:**

The prevalence of age-related eye disorders is increasing with the aging of the global population. Community-based visual health education for the elderly has become a crucial intervention. With the advancement of technology, the application of extended reality (XR), such as virtual reality (VR) and augmented reality (AR), in health education has become more popular. This study aims to assess the effectiveness of educating the elderly about common age-related eye disorders through a novel AR-based health education workshop.

**Methods:**

An AR-based education workshop was designed for the elderly to understand the major visual symptoms of several eye diseases and experience the challenges faced by visually impaired people. The effectiveness of the workshop was assessed by conducting pre- and post-activity surveys to measure the knowledge acquisition of the participants from this workshop.

**Results:**

The intervention was found to significantly improve knowledge of age-related eye diseases among the elderly, while the participants’ age and education level could influence the effectiveness of their knowledge gained from the workshop.

**Conclusions:**

Our study revealed the potential of the use of AR technology in facilitating health education on eye diseases in the elderly. The specific backgrounds and characteristics of target participants and the combination of AR with other pedagogical approaches warrant further investigation to maximize the impact of AR-based workshops in health education in broader healthcare contexts.

**Supplementary Information:**

The online version contains supplementary material available at 10.1186/s12877-024-05658-y.

## Background

Population aging is a pressing global concern, with the proportion of the global elderly population projected to grow from 9.7% in 2022 to 16.4% in 2050 [1]. The increased healthcare burden caused by the aging population will require greater emphasis on prevention and patient education beyond a hospital-based acute care system [2]. Visual impairment is one of the diseases that increases significantly with age, irrespective of other socioeconomic factors [3]. Globally, the leading causes of moderate to severe visual impairment are uncorrected refractive error, cataracts, age-related macular degeneration (AMD), and diabetic retinopathy (DR) [4]. This highlights the need to promote the prevention and education of common age-related eye diseases among the elderly.

Visual disability is perceived to impact various aspects of quality of life, from basic self-care abilities to financial situations and social life. Blindness has been identified as the worst possible ailment, coming before the loss of a limb, deafness, and other devastating ailments. Despite public concerns over the general impacts of visual disability on people’s daily life functions, the elderly population has limited knowledge and awareness of different types of eye diseases associated with visual disability [5]. The mainstream pedagogical methods involve utilizing pamphlets and governmental promotions to raise general awareness. However, these approaches lack the first-hand perception of symptoms of disorders, hence hindering their effectiveness.

Instead, the role of gerontechnology, the combination of gerontology and technology, in promoting awareness of ophthalmic disorders among the elderly should be explored. In particular, the use of extended reality (XR), including augmented reality (AR) and virtual reality (VR), is highly relevant to enhancing visual experiences. AR is a visualization system that merges digital, virtual entities with real-world surrounding environments, allowing users to interactively engage with the contents [6]. It represents a link between the real and digital world in the virtuality continuum [7] and has been frequently used in the field of geriatrics in recent years, such as for balance [8] and cognitive-motor intervention training [9] in the elderly. Moreover, AR technology also demonstrates its strength in educational purposes with the potential to involve gamification elements and promote intergenerational educational experiences for elderly learning, which generally focuses on individual interests and social interaction [10]. Previous studies have shown effectiveness in using XR to facilitate health training and education among the elderly for various purposes, such as oral health education [11], physical exercise [12], and cognitive training [13], suggesting its potential to enhance health awareness among elderly.

The unique potential of AR in visual simulation has prompted its implementation in ophthalmology to raise awareness of common visual disorders. Some AR/VR-based visual defect simulation tools have been previously developed for educational purposes and for identifying accessibility barriers in product and environment designs for individuals with visual impairments [14–17]. However, there has been limited research on how AR can be deployed for visual health education, especially among the elderly population.

In view of the current lack of studies on AR’s roles in elderly visual health education, our study addresses the central question of whether AR technology, integrated with interactive activities simulating daily tasks, can effectively deliver ophthalmic health education to the elderly. Our research aims to study the effectiveness of an AR-based educational workshop utilizing an AR application to educate the elderly on the visual symptoms of common age-related ophthalmic disorders. We hypothesize that this approach will effectively enhance the elderly’s understanding of age-related eye diseases through offering an interactive experience.

## Methods

### Participants

A total of 377 participants aged 60 to 94 (Mean = 73, SD = 8.72) were involved in the study. Elderly subjects aged 60 or above, able to understand Chinese, and mentally competent for informed consent, were recruited from 10 local daily elderly centers voluntarily with informed consent. Those with self-reported severe vision impairment, adverse reactions to AR experiences, or physical or mental illnesses preventing study completion, were excluded.

### Materials

**Fig. 1:**
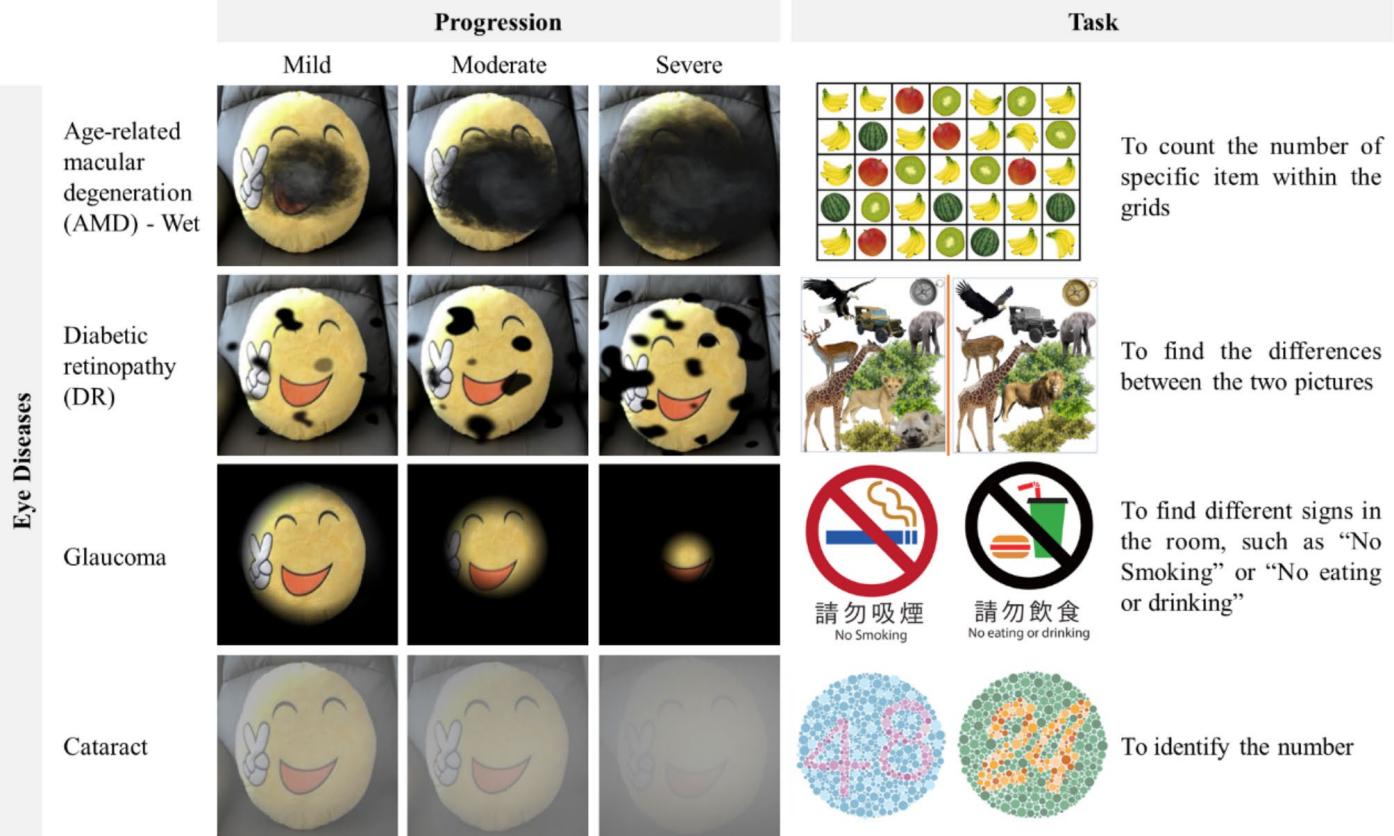
Simulated visual symptoms of different eye diseases at different stages of progression, from mild to severe, and the task of each eye disease for the participants to experience the daily difficulties brought by the corresponding visual symptoms

The AR mobile application, which was developed by the authors, adopts the optical see-through (OST) display technology. The OST display technology allows users to see through the virtual images and maintain their vision of the real environment, offering a high sense of immersion. In our application, virtual images simulating symptoms of various eye diseases were overlayed in users’ field of vision. Three levels of severity (mild, intermediate, severe) are offered to simulate symptoms at different stages of disease progression (Fig. 1).

In this AR-based workshop, four age-related eye diseases were included, namely, age-related macular degeneration (AMD), cataracts, glaucoma and diabetic retinopathy (DR). These eye diseases are four of the leading causes of moderate to severe age-related vision impairment globally [4]. In the AR mobile application, AMD features central vision loss, with an increase in the area of vision loss with increasing severity and wavy vision. Cataract features blurry vision, with increased blurriness with increasing severity. Glaucoma features loss of peripheral vision, with an increase in the area of vision loss towards the central vision with increasing severity. DR features the presence of dots and patches across the visual field, with an increased number of dots and patches with increasing severity.

**Fig. 2:**
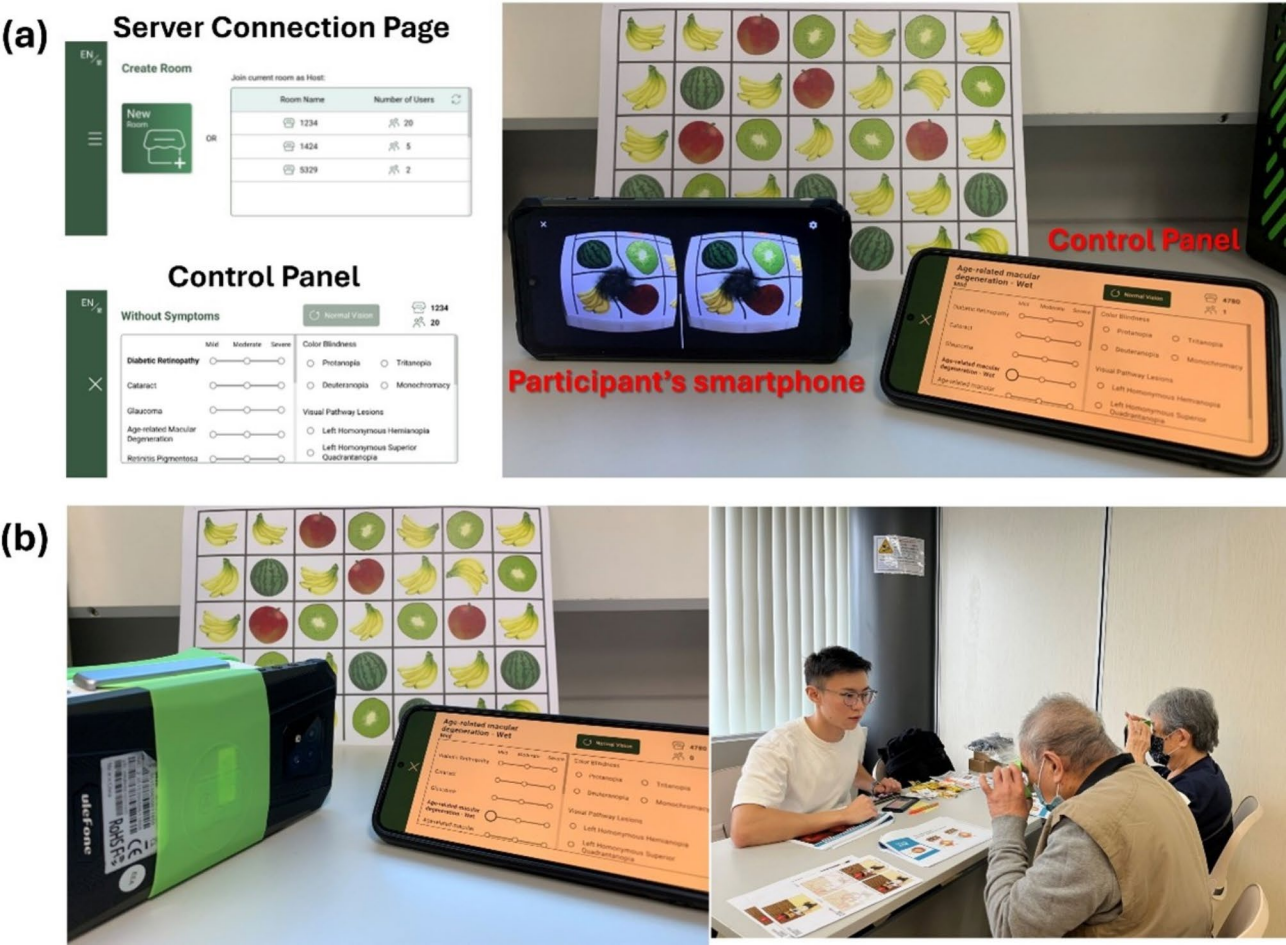
Setup of the AR device. (**a**) A smartphone can be set up as the controller by creating a server using the mobile application interface. The participants’ smartphones can then be connected to the created server, enabling the workshop instructor to apply simulated visual defects to their devices using the control panel interface. (**b**) The participants’ smartphones are mounted on commercially available AR headsets. The elderly participants of the workshop can then hold the device and experience the simulated visual defects

Our own-developed AR mobile application was first installed on smartphones. A head-mounted display (HMD) is adopted, with each smartphone mounted on a commercially available AR headset. With the visual simulation projected through the smartphone-linked headset, users can appreciate the impacts of various eye diseases on their vision. The remote-control function of the application allows the simultaneous control of multiple smartphones on one device to switch between the simulations for the participants (Fig. 2).

### Procedures

Pre-workshop (Supplementary Appendix 1) and post-workshop (Supplementary Appendix 2) questionnaires have been developed for this study.

The participants were then given the pre-workshop questionnaire to collect their sociodemographic information, including gender, age, and education level, together with a test to assess their baseline knowledge of the visual symptoms of four eye diseases, namely AMD, cataract, glaucoma, and DR, on a 5-point scale.

For each disease, our activity began with a 2-minute introduction of the pathophysiology of the disease, followed by a 3-minute task. The participants were provided with the AR headset with the mobile device mounted on and the AR mobile application turned on. Our participants were asked to complete visual-search and visual-discrimination tasks specifically designed for each of the four eye diseases (Fig. 1) while wearing the AR headset to experience how the four eye diseases affect the daily living of people with these eye diseases. Following the completion of each task, the participants were given a 1-2-min debriefing.

After finishing the activities for all four diseases, the participants were asked to complete the post-workshop questionnaire to evaluate their knowledge gain in the four eye diseases after the AR-based workshop.

### Rationales behind the tasks designed

Tasks specific to the four eye diseases were designed for participants to complete while wearing the AR headset to simulate the major symptoms of the diseases and the daily challenges encountered by the patients with the diseases.

For AMD, participants were asked to count the number of specific items segregated in grids on a piece of paper. As AMD features central vision loss and wavy vision, participants would experience difficulties in visualizing items located at the centre. Moreover, they would see curved gridlines, which were very annoying. With the progression of the disease, the area of loss of vision would increase, and participants would experience greater difficulty in locating all their target items across the paper and must increase head movements to facilitate their visualization of the entire paper.

For DR, participants were asked to find the differences between two pictures, with different components scattered across the pictures. As the major symptom of DR is the presence of dots and patches across the visual field, participants could not easily compare the two pictures at the same time without frequent head and eye movements between the two pictures. With an increased number of dots and patches under increasing severity, it would take longer for participants to spot the minute differences between the pictures.

For glaucoma, participants were asked to find different signs posted on the walls of the room, such as “No Smoking” or “No eating or drinking”. As the peripheral vision is lost in glaucoma, participants could only focus on a small area at a time and moved their heads slowly to search for the signs at their seats. Their ability to search was significantly reduced due to the restricted visual field of simulated glaucoma-like vision.

For cataract, participants were asked to identify the numbers shown on Ishihara colour plates. Cataract features blurry vision, and participants would experience challenges in differentiating the borders of the numbers from the background of the Ishihara colour plates. Their ability to perceive colour contrast was reduced under the simulated cataract-like vision. With increased blurriness in more severe modes, participants would find it more difficult to identify the numbers on plates with less colour contrast between the numbers and the background.

### Measurements

The pre-workshop and post-workshop tests each consisted of twenty questions related to the symptoms of the four eye diseases included in the study, and the participants scored 1 point for each correct question. Five questions were asked on each disease, asking participants to determine whether the named symptoms were present in the given disease. Each participant could score a maximum of 5 points for each disease. The post-workshop test shared the same questions as the pre-workshop test. The questions were designed by tertiary educators of ophthalmology, who possess the expertise to determine the knowledge of the eye diseases relevant and significant to the public. The answers of the pre-workshop test were not revealed to the participants until they finished the post-workshop test so that participants could not memorize the correct answers to answer the post-workshop test. This ensured that any increase in knowledge demonstrated from the post-workshop test was due to the knowledge gained through the AR-based workshop.

### Statistics and mathematical analyses

Paired t-tests were performed for each eye disease to evaluate participants’ gain in knowledge of the visual symptoms of the four eye diseases included in this study after the AR-based workshop. Pearson pairwise correlation analysis was conducted to examine whether there were any correlations between their sociodemographic information and their change in score for each disease in the pre- and post-workshop tests. Multiple regression analyses were performed to evaluate the influence of pre-workshop test scores and confounding variables, including gender, age, and education level, on post-workshop test scores. Dummy codes were assigned for the parameter ‘gender’ (male = 1; female = 2) and ‘education level’ (Not know=., No schooling/kindergarten = 0, No formal education = 1, Primary level = 2, Form 3 level = 3, Secondary level = 4, Post-secondary level = 5, University level = 6, Master level and above = 7) in our analyses.

## Results

### Knowledge acquisition

The scores attained by the participants for all four diseases showed a statistically significant increase in the post-workshop test compared with the pre-workshop test (*P* < 0.001, paired t-test), with a large effect for all four diseases (Cohen’s d values > 0.8) (Table 1). This demonstrated that the participants in general had a statistically significant gain in knowledge about the visual symptoms of the four eye diseases.

**Table 1:** Results of paired t-tests between pre- and post-tests of the four eye diseases (*N* = 377)

	Mean change in score (post – pre) [95% CI]	SD	SE mean	t	df	*p*-value	Cohen’s d [95% CI]	Hedge’s correction [95% CI]
AMD	2.451 [2.299 to 2.603]	1.503	0.077	31.663	376	< 0.0001	1.631 [1.476 to 1.784]	1.627 [1.473 to 1.781]
Cataract	1.35 [1.156 to 1.544]	1.916	0.099	13.685	376	< 0.0001	0.705 [0.592 to 0.817]	0.703 [0.590 to 0.816]
Glaucoma	2.562 [2.389 to 2.736]	1.713	0.088	29.051	376	< 0.0001	1.496 [1.349 to 1.643]	1.493 [1.346 to 1.639]
DR	2.432 [2.28 to 2.585]	1.508	0.078	31.328	376	< 0.0001	1.613 [1.460 to 1.766]	1.610 [1.457 to 1.763]

### Correlation analysis

The analysis showed a negative correlation between the age of the participants and knowledge gain in glaucoma and DR (*P* < 0.001, paired t-test) but no significant correlation for AMD and cataract (Table 2). Meanwhile, a positive correlation was found between the education level of the participants and knowledge gain in glaucoma and DR (*P* < 0.05 and *P* < 0.001 respectively, paired t-test) but no significant correlation for AMD and cataract (Table 2). There is no significant correlation between gender and participants’ knowledge gain in AMD, cataract and glaucoma (Table 2). This shows that the age and education level of participants can limit their knowledge acquisition from the workshop to a certain extent, suggesting that it is a factor to consider but not a major hindrance in the future application of our AR-based workshop.

**Table 2:** Correlations between the gender, age and education level of the participants and the change in scores for the four eye diseases in the pre- and post-workshop tests (*N* = 377)

		Gender	Age	Education level	Change in score (AMD)	Change in score (Cataract)	Change in score (Glaucoma)
Gender	Pearson correlation coefficient [95% CI]	-	-	-	-	-	-
	p-value	-	-	-	-	-	-
Age	Pearson correlation coefficient [95% CI]	-0.019 [-0.119 to 0.082]	-	-	-	-	-
	p-value	0.7173	-	-	-	-	-
Education Level	Pearson correlation coefficient [95% CI]	0.179 [0.077 to 0.277]	-0.463 [-0.540 to -0.378]	-	-	-	-
	p-value	0.0006	< 0.0001	-	-	-	-
Change in score (AMD)	Pearson correlation coefficient [95% CI]	0.054 [-0.470 to 0.154]	-0.083 [-0.183 to 0.018]	0.034 [-0.070 to 0.137]	-	-	-
	p-value	0.2942	0.1065	0.5219	-	-	-
Change in score (Cataract)	Pearson correlation coefficient [95% CI]	0.047 [-0.055 to 0.147]	-0.058 [-0.158 to 0.044]	0.001 [-0.103 to 0.104]	0.170 [0.071 to 0.267]	-	-
	p-value	0.3669	0.2645	0.9921	0.0009	-	-
Change in score (Glaucoma)	Pearson correlation coefficient [95% CI]	0.056 [-0.045 to 0.156]	-0.209 [-0.304 to -0.110]	0.135 [0.032 to 0.235]	0.247 [0.150 to 0.340]	0.269 [0.173 to 0.360]	-
	p-value	0.2796	< 0.0001	0.0103	< 0.0001	< 0.0001	-
Change in score (DR)	Pearson correlation coefficient [95% CI]	0.146 [0.045 to 0.243]	-0.222 [-0.316 to -0.124]	0.223 [0.122 to 0.319]	0.123 [0.022 to 0.221]	0.139 [0.039 to 0.237]	0.322 [0.228 to 0.409]
	p-value	0.0046	< 0.0001	< 0.0001	0.0172	0.0069	< 0.0001

### Multiple regressions

After controlling the effects of gender, age, and education level, only pre-workshop test scores of cataract and glaucoma significantly predicted their corresponding post-workshop test scores (Tables 4 and 5, *P* < 0.0001 for cataract and *P* < 0.1 for glaucoma). Gender positively predicted the post-workshop test score of DR (Table 6, *P* < 0.1). Age negatively predicted the post-workshop test score of AMD, glaucoma and DR (Tables 3, 5 and 6, *P* < 0.001 for AMD, *P* < 0.0001 for glaucoma, and *P* < 0.001 for DR). Education level positively predicted all four post-workshop test scores (Tables 3, 4, 5, and 6, *P* < 0.001 for AMD, *P* < 0.0001 for cataract, *P* < 0.001 for glaucoma, and *P* < 0.01 for DR). These results suggest that (1) participants who got higher scores in the pre-workshop test were more likely to get higher scores in the post-workshop test for cataract and glaucoma; (2) female participants are more likely to get higher scores in the post-workshop test of DR; and (3) participants are generally more likely to get higher scores if they are younger and more educated.

**Table 3:** Multiple regression on post-workshop test score of AMD (*N* = 377) (adjusted R Square = 0.110, *p* = 3.0916E-9)

	Unstandardized B [95% CI]	Standard error	Standardized coefficient 𝛽	t	*p*-value
Pre-workshop test score	-0.007 [-0.102 to 0.088]	0.048	-0.007	-0.146	0.8842
Gender (Male = 1; Female = 2)	0.174 [-0.098 to 0.446]	0.138	0.064	1.259	0.2088
Age	-0.027 [-0.042 to -0.011]	0.008	-0.193	-3.435	0.0007
Education level	0.147 [0.062 to 0.233]	0.043	0.197	3.393	0.0008

**Table 4:** Multiple regression on post-workshop test score of cataract (*N* = 377) (adjusted R Square = 0.196, *p* = 7.1121E-17)

	Unstandardized B [95% CI]	Standard error	Standardized coefficient 𝛽	t	*p*-value
Pre-workshop test score	0.200 [0.130 to 0.269]	0.035	0.277	5.656	< 0.0001
Gender	0.114 [-0.212 to 0.441]	0.166	0.033	0.689	0.4913
Age	-0.015 [-0.033 to 0.004]	0.009	-0.085	-1.582	0.1145
Education Level	0.226 [0.123 to 0.330]	0.053	0.240	4.308	< 0.0001

**Table 5:** Multiple regression on post-workshop test score of glaucoma (*N* = 377) (adjusted R Square = 0.153, *p* = 6.2941E-13)

	Unstandardized B [95% CI]	Standard error	Standardized coefficient 𝛽	t	*p*-value
Pre-workshop test score	0.156 [0.021 to 0.291]	0.069	0.114	2.268	0.0239
Gender	0.06 [-0.321 to 0.441]	0.193	0.015	0.310	0.7567
Age	-0.045 [-0.066 to -0.023]	0.011	-0.225	-4.110	< 0.0001
Education Level	0.209 [0.089 to 0.329]	0.061	0.195	3.424	0.0007

**Table 6:** Multiple regression on post-workshop test score of DR (*N* = 377) (adjusted R Square = 0.115, *p* = 1.0462E-9)

	Unstandardized B [95% CI]	Standard error	Standardized coefficient 𝛽	t	*p*-value
Pre-workshop test score	0.035 [-0.190 to 0.260]	0.114	0.015	0.304	0.7613
Gender	0.41 [0.050 to 0.770]	0.183	0.113	2.242	0.0256
Age	-0.041 [-0.061 to -0.020]	0.010	-0.221	-3.927	0.0001
Education Level	0.152 [0.040 to 0.260]	0.057	0.153	2.686	0.0076

## Discussion

### Principal findings

The current study investigated the impact of an AR-based health education workshop on knowledge acquisition in eye diseases among the elderly in a quasi-experimental setting. Paired t-tests demonstrated that the AR-based workshop significantly increased our elderly participants’ knowledge of various ophthalmic diseases after their experience of the visual symptoms. This suggested that the integration of AR into health education initiatives for the elderly can facilitate their comprehension of eye diseases and the associated challenges faced by patients with these diseases. This would help them appraise the significance of visual health and draw their attention to their eye conditions more effectively.

Our study also identified a negative correlation between participants’ age and their knowledge gain while a positive correlation between their education level and their knowledge gain in some diseases. This indicated that the effectiveness of the AR-based workshop on the knowledge acquisition of participants might differ between elderly participants from different backgrounds, and modifications might be required to tailor the learning experience for different groups of participants.

### Significance

Visual health is integral not only to the quality of life but also to the elimination of numerous social problems, drawing mounting attention to the significance of visual health education within the community [18]. While elderly participants are more likely to have a medical history of eye diseases, a study showed that having a related medical history negatively correlates with the knowledge level of eye diseases [5], highlighting the significance of further raising the understanding of eye diseases among the elderly population through health education workshops.

Previous studies have elucidated the potential roles of XR in various aspects of ophthalmology, including education, diagnostics and therapeutics [19], demonstrating how the visual features of XR make it highly relevant and applicable to the ophthalmology specialty. A study has further highlighted the potential of XR for scalable health promotion, where participants demonstrated an improved understanding of eye diseases and appraised the significance of eye screening after utilizing XR tools [20], suggesting the effectiveness of XR in raising the general public’s awareness of the significance of visual health. In addition to its educational value, XR-based educational models have been demonstrated to offer highly acceptable, safe, engaging, and enjoyable experiences for both youngsters and adults [21, 22]. Several XR systems, such as SIMVIZ [23], OpenVisSim [14, 15], and CatARact [16]. These studies have illustrated the strengths of AR in simulating the visual symptoms of various ophthalmic disorders and offering an immersive, simulative experience for the users. Our study further demonstrated the application of AR in visual health education was effective in the elderly group.

Our team has also attempted to conduct the AR-based workshop for educating medical students and high school students on common eye disorders. Beyond knowledge acquisition, the AR intervention was found effective in cultivating a high level of empathy. Apart from describing the visual impairments in different diseases, the participants could contextualize them with possible difficulties in patients’ daily lives. This reflects the suitability of this intervention for different participant groups and its potential application for broader purposes, such as training caregivers of patients with eye diseases or future healthcare practitioners. The participants also reported that the AR intervention was a delightful and meaningful experience, which enhanced their curiosity about various eye diseases. It suggests that the AR intervention can serve as a sustainable educational approach, motivating participants to actively acquire health knowledge, thus demonstrating its clinical significance in contributing to community-based health promotion and preventive care initiatives. Expanding the application of such an immersive AR simulation experiential learning workshop to different participant groups, diseases and educational purposes can further develop its potential to enhance awareness, facilitate early detection, lifestyle modifications, and patient-centred care.

In our study, a negative correlation existed between age and knowledge gain in glaucoma and DR. Declined cognitive ability as a result of aging [24, 25] might be the reason that older participants might find it harder to retain and process the new information acquired. Alternatively, older participants might be less receptive to learning with AR due to a higher chance of experiencing cyber-sickness [10]. Studies found that AR exposure could lead to symptoms of cyber-sickness, such as oculomotor disturbance, disorientation, and nausea [26]. In our study, participants were not observed to have any significant presentation of symptoms of cyber-sickness. However, further investigation of the effects of prolonged AR exposure in the setting of an interactive workshop is recommended. This also highlights the need to consider the physical and social discrepancies between different age groups when designing AR-based health education workshops.

Conversely, education level positively correlated with knowledge gain in glaucoma and DR. This is unsurprising as participants with a higher education level might have a higher health literacy and thus could grasp new health information more efficiently. This suggests the significance of raising the elderly’s health literacy and the need to tailor technology-based health education according to different learning abilities or education levels.

### Strengths

This study presented an innovative and interactive approach to educate elderly participants on visual health. By overlaying virtual elements in the real-world environment, AR can present complex medical concepts in a simplified and visually engaging manner. Such a visual representation can aid participants’ comprehension, particularly for elderly learners who may have difficulty understanding abstract or complex information through text-based or verbal descriptions alone, while offering them a memorable and enjoyable learning experience.

Besides, the current AR-based workshop involved interactive components. The AR application was coupled with specially designed tasks relevant to the main symptoms of the eye diseases. Unlike the traditional passive, didactic approach, the participants were encouraged to actively explore with the AR headset via experiential learning. Through task completion, participants also participated in problem solving and decision making. These cognitively stimulating activities and the hands-on experience would help participants strengthen their memory of the knowledge gained and maintain their attention, especially for elderly participants who might have a shorter attention span and potentially struggle to stay engaged with traditional instrumental methods.

Apart from the simulative tasks, our application has a remote-control system, which allows our simultaneous control of multiple devices, facilitating our running of group-based workshop or even larger class workshops. With the flexible manipulation of the AR application, the current AR-based workshop can be customized to address specific visual symptoms and levels of severity of common age-related eye diseases, offering personalised education relevant to individuals’ needs. Given the accessibility of the AR headset, prevalence of mobile devices, easy manipulation of the application and portability of the set-up, such an intervention can be widely available in different settings for health education. Apart from community-based health promotion, it can be deployed for educating patients about various visual disorders, complications, and disease progression in a clinical setting [16]; or offering healthcare practitioners insights into patients’ experience of their visual impairments, facilitating informed discussion, and promoting patients’ engagement in visual health management. It can also be used for educating caregivers of patients with eye diseases, helping them experience patients’ visual symptoms and their daily challenges and increasing their empathy.

### Limitations and future improvements

The current study has proven the effectiveness of our AR intervention in facilitating our elderly participants’ learning of eye diseases with a large effect size. However, several limitations were presented. First, our study lacked a control group. Despite the large effect size demonstrated by the statistical analyses, uncertainties remain over whether the AR intervention is more effective than other educational approaches. Further studies should be done to compare the AR-based workshop with other strategies, such as health talks, individual counselling, and other multimedia, such as videos, as well as combined methods that incorporate AR with other approaches, to identify the best way to facilitate visual health education among the elderly. Nevertheless, in our current study, we performed multiple regressions that controlled the effects of confounding factors, including gender, age, and education level of the participants.

Secondly, the pre-workshop and post-workshop tests used were not validated knowledge surveys. This is because the questions involved in the current tests were rather direct and simple. The participants were asked about symptoms of the diseases that were directly simulated by the AR application. Additionally, the test was developed by professionals in the field, so it has faced validity regarding the content. Answers of the test were not revealed to the participants until they finished the post-workshop test. This procedure ensured that the participants did not get a higher score in the post-workshop test by simply memorising the answers, but through our intervention.

The current study aimed to serve as a pilot evaluation of our elderly participants’ performance in acquiring simple basic knowledge from the AR intervention before testing its effectiveness in facilitating the elderly’s acquisition of more advanced knowledge and other aspects of learning effectiveness. Future studies should include more parameters of learning effectiveness, such as content quality, self-efficacy, and application, as well as other parameters related to participants’ learning experience, such as learners’ attitudes towards AR, flexibility, enjoyment and the application’s interface design, considering that some elderly individuals may have limited memory, attention span and executive functions due to declined cognitive ability. This will help evaluate the suitability of an AR intervention in health education targeting the elderly more comprehensively.

Finally, the current study was a one-off intervention. Further investigations should be performed to assess the effectiveness of the AR-based workshop on participants’ long-term knowledge acquisition, especially for the elderly, who might have shorter memory due to declined cognitive functions. Whether the knowledge gained from the AR-based workshop could effectively raise the elderly’s awareness of their own visual health should also be evaluated.

### Future directions

This study has demonstrated the effectiveness of an AR-based workshop in facilitating visual health education in the elderly, and it could potentially be applied in a wider context, such as other aspects of health education beyond visual symptoms of eye diseases, or in other settings beyond community health promotion, such as patient education in clinical settings, caregiver training, or healthcare profession training. Further expansion of the subject recruitment beyond Hong Kong elderly centers can be done to increase the diversity of the participant pool and enhance the generalizability of the intervention across regions. To evaluate the suitability of applying AR in more aspects of elderly health education, apart from evaluating the effectiveness of knowledge acquisition by participants, the user experience in interacting with AR technologies could be further examined to assess the accessibility and acceptability of AR applications by the elderly and identify areas for improvements in the application’s interface design and user guidance during the AR-based workshop. Further studies can focus on exploring methods to tailor the intervention to participants of different ages and education levels. Besides, multimodal approaches involving the combination of AR with other pedagogical strategies, such as gamification, or technologies like VR, can be explored to maximize participants’ learning experience. Finally, a cost-effectiveness analysis can be conducted to evaluate the economic viability of implementing AR-based health education interventions for the elderly population, especially in less developed areas. The potential cost savings, scalability, and sustainability of using AR technologies should be assessed and compared with those of traditional educational methods to evaluate their suitability for large-scale and long-term implementation in community-based health education.

## Conclusion

Our study has unveiled the potential of AR technology in visual health promotion among the elderly. To fully realize the potential of the AR-based workshop, more parameters should be included to evaluate its effectiveness in promoting knowledge acquisition and other aspects contributing to its suitability for the elderly’s usage. The learning abilities, knowledge, and other physical and social backgrounds of participants should be taken into consideration when designing AR-based education workshops to maximize the benefits gained from the interventions. The longer-term effect on the knowledge gained by the elderly through this AR-based health education workshop should also be investigated to better evaluate the impacts of the intervention on raising the health awareness of the elderly. Its application in a broader context in health education and its potential combination with other pedagogical approaches can be further explored to expand its potential.

## Electronic supplementary material

Below is the link to the electronic supplementary material.

Supplementary Material 1

Supplementary Material 2

## Data Availability

Data is provided within the manuscript.

## Abbreviations

XR: Extended reality
VR: Virtual reality
AR: Augmented reality
OST: Optical see-through
HMD: Head-mounted display
AMD: Age-related macular degeneration
DR: Diabetic retinopathy
