# Supplementary material for: The interactive use of augmented reality for educating the elderly on common age-related eye disease
Source: BMC Geriatr. 2025 Jan 3;25:6. doi: 10.1186/s12877-024-05658-y (PMC11697621; doi:10.1186/s12877-024-05658-y)
Supplement: Supplementary file 1 — Supplementary Material 1 [file 12877_2024_5658_MOESM1_ESM.docx]

**Introduction of Augmented Reality (AR) technologies to educate public the visual symptoms and progression of important vision-threatening diseases**

**Post-activity questionnaire – Elderly or caregivers**

Reference number: ___

1. After the event, do you think you understand more about AMD?

❑ Yes

❑ No

1. Will the following problem(s) be seen in AMD patient’s vision? (Refer to the diagram attached)
   1. Affect Central vision

❑ Yes

❑ No

❑ Don’t know

- 1. Wavy vision

❑ Yes

❑ No

❑ Don’t know

- 1. Double Image

❑ Yes

❑ No

❑ Don’t know

2.4 Black patch in Central vision

❑ Yes

❑ No

❑ Don’t know

2.5 Black spots in Peripheral vision

❑ Yes

❑ No

❑ Don’t know

1. After the event, do you think you understand more about Cataract?

❑ Yes

❑ No

1. Will the following problem(s) be seen in Cataract patient’s vision? (Refer to the diagram attached)

4.1 Blurry vision

❑ Yes

❑ No

❑ Don’t know

4.2 Wavy vision

❑ Yes

❑ No

❑ Don’t know

4.3 Floaters

❑ Yes

❑ No

❑ Don’t know

4.4 Black patch in Central vision

❑ Yes

❑ No

❑ Don’t know

4.5 Black spots in Peripheral vision

❑ Yes

❑ No

❑ Don’t know

1. After the event, do you think you understand more about Glaucoma?

❑ Yes

❑ No

1. Will of the following problem(s) be seen in Glaucoma patient’s vision? (Refer to the diagram attached)

6.1 Affect Central vision

❑ Yes

❑ No

❑ Don’t know

6.2 Affect Peripheral vision

❑ Yes

❑ No

❑ Don’t know

6.3 Wavy vision

❑ Yes

❑ No

❑ Don’t know

6.4 Floaters

❑ Yes

❑ No

❑ Don’t know

6.5 Black patch in Central vision

❑ Yes

❑ No

❑ Don’t know

1. After the event, do you think you understand more about Diabetic Retinopathy?

❑ Yes

❑ No

1. Will the following problem(s) be seen in Diabetic Retinopathy patient’s vision? (Refer to the diagram attached)

8.1 Affect Peripheral vision

❑ Yes

❑ No

❑ Don’t know

8.2 Double image

❑ Yes

❑ No

❑ Don’t know

8.3 Black patch in Central vision

❑ Yes

❑ No

❑ Don’t know

8.4 Black spots in Peripheral vision

❑ Yes

❑ No

❑ Don’t know

8.5 Increase number of black spots

❑ Yes

❑ No

❑ Don’t know

Attachment:


**《藉增強現實(AR)技術教育公眾常見眼疾的視覺症狀及疾病進展》**

**活動後問卷 – 長者或照顧者**

編號 : ___

1. 活動後，你對老年黃斑病變有沒有更深刻的認識？

❑有

❑ 沒有

1. 老年黃斑病變患者的視力會否出現以下這些問題？（參考附件一作答）

2．1 影響中央視力

❑會

❑ 不會

❑不知道

2．2 影像變形彎曲

❑會

❑ 不會

❑不知道

2．3 雙重影像

❑會

❑ 不會

❑不知道

2．4 視野中央出現黑塊

❑會

❑ 不會

❑不知道

2．5 周圍出現斑點

❑會

❑ 不會

❑不知道

1. 活動後，你對白內障有沒有更深刻的認識？

❑有

❑ 沒有

1. 白內障患者的視力會否出現以下這些問題？（參考附件一作答）

4．1 視力模糊

❑會

❑ 不會

❑不知道

4．2 影像變形彎曲

❑會

❑ 不會

❑不知道

4．3 飛蚊症

❑會

❑ 不會

❑不知道

4．4 視野中央出現黑塊

❑會

❑ 不會

❑不知道

4．5 周圍出現斑點

❑會

❑ 不會

❑不知道

1. 活動後，你對青光眼有沒有更深刻的認識？

❑有

❑ 沒有

1. 青光眼患者的視力會否出現以下這些問題？（參考附件一作答）

6．1 影響中央視力

❑會

❑ 不會

❑不知道

6．2 影響周邊視力

❑會

❑ 不會

❑不知道

6．3 影像變形彎曲

❑會

❑ 不會

❑不知道

6．4 飛蚊症

❑會

❑ 不會

❑不知道

6．5 視野中央出現黑塊

❑會

❑ 不會

❑不知道

1. 活動後，你對糖尿病視網膜病變有沒有更深刻的認識？

❑有

❑ 沒有

1. 糖尿病視網膜病變患者的視力會否出現以下這些問題？（參考附件一作答）

8．1 影響周邊視力

❑會

❑ 不會

❑不知道

8．2 雙重影像

❑會

❑ 不會

❑不知道

8．3 視野中央出現黑塊

❑會

❑ 不會

❑不知道

8．4周圍出現斑點

❑會

❑ 不會

❑不知道

8．5 斑點數量增加

❑會

❑ 不會

❑不知道

附件一：
