# Supplementary material for: The interactive use of augmented reality for educating the elderly on common age-related eye disease
Source: BMC Geriatr. 2025 Jan 3;25:6. doi: 10.1186/s12877-024-05658-y (PMC11697621; doi:10.1186/s12877-024-05658-y)
Supplement: Supplementary file 2 — Supplementary Material 2 [file 12877_2024_5658_MOESM2_ESM.docx]

**Introduction of Augmented Reality (AR) technologies to educate public the visual symptoms and progression of important vision-threatening diseases**

**Pre-activity questionnaire – Elderly or caregivers**

Reference number: ___

**Personal Information**

1. Gender: ❑Male ❑Female
2. Age: ___
3. Please tell me your highest educational attainment

❑ Master level or above

❑ University level (Degree)

❑ Post-secondary level (Non-degree)

❑ Secondary level (Incl. Form 6 and Form 7)

❑ Form 3 level

❑ Primary level

❑ No formal education (Can read newspaper and write)

❑ No schooling/ kindergarten

❑ Not known

1. Have you ever suffered from the following diseases?

❑ Diabetes Mellitus

❑ Hypertension

❑ Hyperlipidemia

❑ Glaucoma

❑ Cataract

❑ Age-related Macular Degeneration (AMD)

❑ Diabetic retinopathy

❑ Colour blind

❑ None of the above

1. Have your family members (including parents, spouse and children) ever suffered from the following diseases?

❑ Diabetes Mellitus

❑ Hypertension

❑ Hyperlipidemia

❑ Glaucoma

❑ Cataract

❑ Age-related Macular Degeneration (AMD)

❑ Diabetic retinopathy

❑ None of the above

**Knowledge related to Age-related Macular Degeneration**

1. Have you heard of Age-related Macular Degeneration (AMD)?

❑ Yes

❑ No (If no, skip to Cataract)

1. Do you know what is/are the risk factor(s) for AMD? If yes, please list them out below.
2. Do you know some visual symptoms of AMD?

❑ Yes (If Yes, Will the following problem(s) be seen in AMD patient’s vision? (Refer to the diagram attached))

❑ No (If no, please skip to Q9)

- 1. Affect Central vision

❑ Yes

❑ No

❑ Don’t know

- 1. Wavy vision

❑ Yes

❑ No

❑ Don’t know

- 1. Double Image

❑ Yes

❑ No

❑ Don’t know

8.4 Black patch in Central vision

❑ Yes

❑ No

❑ Don’t know

8.5 Black spots in Peripheral vision

❑ Yes

❑ No

❑ Don’t know

1. Do you know what is/are the treatment(s) for AMD? If yes, please list them out below.

**Knowledge related to Cataract**

1. Have you heard of the Cataract?

❑ Yes

❑ No (If no, skip to Glaucoma)

1. Do you know what is/are the risk factor(s) for Cataract? If yes, please list them out below.
2. Do you know some visual symptoms of Cataract?

❑ Yes (If Yes, Will the following problem(s) be seen in Cataract patient’s vision? (Refer to the diagram attached))
❑ No (If no, please skip to Q13)

12.1 Blurry vision

❑ Yes

❑ No

❑ Don’t know

12.2 Wavy vision

❑ Yes

❑ No

❑ Don’t know

12.3 Floaters

❑ Yes

❑ No

❑ Don’t know

12.4 Black patch in Central vision

❑ Yes

❑ No

❑ Don’t know

12.5 Black spots in Peripheral vision

❑ Yes

❑ No

❑ Don’t know

1. Do you know what is/are the treatment(s) for Cataract? If yes, please list them out below.

**Knowledge related to Glaucoma**

1. Have you heard of the Glaucoma?

❑ Yes

❑ No (If no, skip to Diabetic Retinopathy)

1. Do you know what is/are the risk factor(s) for Glaucoma? If yes, please list them out below.
2. Do you know some visual symptoms of Glaucoma?

❑ Yes (If Yes, Will of the following problem(s) be seen in Glaucoma patient’s vision? (Refer to the diagram attached))

❑ No (If no, please skip to Q17)

16.1 Affect Central vision

❑ Yes

❑ No

❑ Don’t know

16.2 Affect Peripheral vision

❑ Yes

❑ No

❑ Don’t know

16.3 Wavy vision

❑ Yes

❑ No

❑ Don’t know

16.4 Floaters

❑ Yes

❑ No

❑ Don’t know

16.5 Black patch in Central vision

❑ Yes

❑ No

❑ Don’t know

1. Do you know what is/are the treatment(s) for Glaucoma? If yes, please list them out below.

**Knowledge related to Diabetic Retinopathy**

1. Have you ever heard of Diabetic Retinopathy?

❑ Yes

❑ No (If no, the questionnaire ends)

1. Do you know what is/are the risk factor(s) for Diabetic Retinopathy? If yes, please list them out below
2. Do you know some visual symptoms of Diabetic Retinopathy?

❑ Yes (If Yes, Will the following problem(s) be seen in Diabetic Retinopathy patient’s vision? (Refer to the diagram attached))

❑ No (If no, please skip to Q21)

20.1 Affect Peripheral vision

❑ Yes

❑ No

❑ Don’t know

20.2 Double image

❑ Yes

❑ No

❑ Don’t know

20.3 Black patch in Central vision

❑ Yes

❑ No

❑ Don’t know

20.4 Black spots in Peripheral vision

❑ Yes

❑ No

❑ Don’t know

20.5 Increase number of black spots

❑ Yes

❑ No

❑ Don’t know

1. Do you know what is/are the treatment(s) for Diabetic Retinopathy? If yes, please list them out below.

Attachment:


**《藉增強現實(AR)技術教育公眾常見眼疾的視覺症狀及疾病進展》**

**活動前問卷 – 長者或照顧者**

編號 : ___

**個人資料**

1. 性別 ❑男 ❑女
2. 年齡: ___
3. 您的教育程度是 :

❑碩士或以上

❑大學 (學位)

❑專上 (非學位)

❑中學 (包括預科程度)

❑初中

❑小學

❑未受正規教育 (能閱讀報紙及寫信)

❑未受教育/幼稚園
❑不知道

1. 你曾否患有以下疾病？

❑糖尿病

❑高血壓

❑高血脂

❑ 青光眼

❑白內障

❑老年黃斑病變

❑糖尿病視網膜病變

❑色盲

❑以上都不是

1. 你的家人（包括父母、配偶及子女）曾否患有以下的疾病？

❑糖尿病

❑高血壓

❑高血脂

❑ 青光眼

❑白內障

❑老年黃斑病變

❑糖尿病視網膜病變

❑以上都不是

**對老年黃斑病變(AMD)的認知**

1. 你有聽過老年黃斑病變(AMD)嗎？

❑有

❑ 沒有 （如沒有，直接跳至白內障）

1. 你知道有什麼因素會提高患上老年黃斑病變的風險嗎？如知道，請在下面列出。
2. 你知道一些老年黃斑病變的病徵嗎？

❑知道 （如知道，老年黃斑病變患者的視力會否出現以下這些問題？（參考附件一作答））

❑ 不知道（如不知道，直接跳至第9題）

8．1 影響中央視力

❑會

❑ 不會

❑不知道

8．2 影像變形彎曲

❑會

❑ 不會

❑不知道

8．3 雙重影像

❑會

❑ 不會

❑不知道

8．4 視野中央出現黑塊

❑會

❑ 不會

❑不知道

8．5 周圍出現斑點

❑會

❑ 不會

❑不知道

1. 你知道有什麼治療老年黃斑病變的方法嗎? 如知道，請在下面列出。

**對白內障的認知**

10. 你有聽過白內障嗎？

❑有

❑ 沒有 （如沒有，直接跳至青光眼）

11．你知道有什麼因素會提高患上白內障的風險嗎？如知道，請在下面列出。

12． 你知道一些白內障的病徵嗎？

❑知道 （如知道，白內障患者的視力會否出現以下這些問題？（參考附件一作答））

❑ 不知道（如不知道，直接跳至第13題）

12．1 視力模糊

❑會

❑ 不會

❑不知道

12．2 影像變形彎曲

❑會

❑ 不會

❑不知道

12．3 飛蚊症

❑會

❑ 不會

❑不知道

12．4 視野中央出現黑塊

❑會

❑ 不會

❑不知道

12．5 周圍出現斑點

❑會

❑ 不會

❑不知道

13． 你知道有什麼治療白內障的方法嗎? 如知道，請在下面列出。

**對青光眼的認知**

14. 你有聽過青光眼嗎？

❑有

❑ 沒有 （如沒有，直接跳至糖尿病視網膜病變）

15．你知道有什麼因素會提高患上青光眼的風險嗎？如知道，請在下面列出。

16. 你知道一些青光眼的病徵嗎？

❑知道 （如知道，青光眼患者的視力會否出現以下這些問題？（參考附件一作答））

❑ 不知道（如不知道，直接跳至第17題）

16．1 影響中央視力

❑會

❑ 不會

❑不知道

16．2 影響周邊視力

❑會

❑ 不會

❑不知道

16．3 影像變形彎曲

❑會

❑ 不會

❑不知道

16．4 飛蚊症

❑會

❑ 不會

❑不知道

16．5 視野中央出現黑塊

❑會

❑ 不會

❑不知道

17．你知道有什麼治療青光眼的方法嗎? 如知道，請在下面列出。

**對糖尿病視網膜病變的認知**

18. 你有聽過糖尿病視網膜病變嗎？

❑有

❑ 沒有 （如沒有，問卷結束）

19. 你知道有什麼因素會提高患上糖尿病視網膜病變的風險嗎？如知道，請在下面列出。

20. 你知道一些糖尿病視網膜病變的病徵嗎？

❑知道 （如知道，糖尿病視網膜病變患者的視力會否出現以下這些問題？（參考附件一作答））

❑ 不知道（如不知道，直接跳至第21題）

20．1 影響周邊視力

❑會

❑ 不會

❑不知道

20．2 雙重影像

❑會

❑ 不會

❑不知道

20．3 視野中央出現黑塊

❑會

❑ 不會

❑不知道

20．4周圍出現斑點

❑會

❑ 不會

❑不知道

20．5 斑點數量增加

❑會

❑ 不會

❑不知道

21．你知道有什麼治療糖尿病視網膜病變的方法嗎? 如知道，請在下面列出 。

附件一：
